# Providers’ Stigmas and the Effects on Patients with Opioid Use Disorder: A Scoping Review

**DOI:** 10.13023/jah.0403.06

**Published:** 2023-01-01

**Authors:** Peyton Skaggs, Sarah Beth Bell, F. Douglas Scutchfield, Lauren E. Robinson

**Affiliations:** University of Kentucky, peytonskaggs@uky.edu; University of Oklahoma; University of Kentucky; University of Kentucky

**Keywords:** Appalachia, Opioid Use Disorder, Stigma, bias, healthcare barriers

## Abstract

**Introduction:**

One of the most prevalent, dangerous stigmas in health care is the complex bias toward patients with opioid use disorder (OUD). This stigma damages the vital patient–provider relationship, further perpetuating the opioid epidemic.

**Purpose:**

Unfortunately, research on the relationship between OUD and provider stigma is greatly lacking. To fill this gap, the present in-depth study undertakes a scoping review of research on providers’ stigma toward OUD in order to determine how enacted stigma affects treatment plans.

**Methods:**

Four databases were used to identify articles published from 1999 to 2021. A comprehensive search strategy was developed through a collaborative process between the researchers and a medical librarian. The researchers used the methodological framework developed by Arksey and O’Malley (2005) and expanded upon by Levac et al.(2010) to chart study characteristics and themes.

**Results:**

A total of 196 search items were retrieved. After de-duplication (n=31), remaining articles were screened based on the inclusion and exclusion criteria detailed in the protocol. After both a title/abstract review and full-text review, an additional 158 articles were removed. This yielded a total of seven articles. Three main themes were identified in the literature: (1) rural–urban differences in bias; (2) provider concern regarding legal implications and regulatory concerns; and (3) the belief that OUD is a moral failing rather than a medical diagnosis.

**Implications:**

Additional research should further analyze prescribed treatment plans for patients with OUD and utilize this information to create future considerations aimed at reducing opioid-related stigma in healthcare in Appalachia.

## INTRODUCTION

Appalachia leads the country in both mortality and morbidity, and a variety of health outcomes remain far below the national average.^1^ In Appalachian counties, accident-prone industries, such as coal mining, account for over 25% of total employment.^2^ High rates of opioid use disorder (OUD) in Appalachia are thought to be a byproduct of the region’s high level of injury-prone employment, historic overprescribing of opioids, and lack of community health services.^1^ The region’s abundant availability of opioids has remained consistent over the past decade, with opioid prescription rates 45% higher in Appalachian counties than in the remainder of the country.^2^ The mass volume of both prescription and illicit opioids has left the region exceptionally susceptible to opioid misuse.

Adding to the region’s vulnerability, opioid-related stigma—a complex, negative attitude toward people who use opioids—worsens both healthcare access and quality of care. This stigma remains largely unaddressed in Appalachia, and the lack of scrutiny regarding this substantial bias proves to be a missed opportunity for significantly improving Appalachian health care. Isolated research studies focused on the relationship between OUD and provider stigma on their own are insufficient in understanding the totality of enacted opioid-related stigma, creating the need for this in-depth analysis.

Amidst this context, the following research question led this scoping review: What are providers’ stigmas and prescribed treatment plans for those with OUD in Appalachia? In answer, the research team investigated and systematically mapped the evidence available in peer-reviewed literature in order to better understand the ways provider bias manifests into enacted stigma.

### Study Objectives

This scoping review offers a comprehensive literature review of included articles focused on provider bias towards patients with OUD in Appalachia. The objectives of this review are to (1) analyze and summarize the results of peer-reviewed literature examining stigma associated with OUD and how these stigmas affect providers’ prescribed treatment plans; (2) categorize these factors and investigate developing patterns; (3) provide recommendations aimed at decreasing stigma associated with OUD to prevent bias in providers’ treatment plans; and (4) based on gaps in the literature, determine areas in which more research is necessary.

## METHODS

A scoping review was conducted to examine the existing literature on the role of healthcare providers’ stigmas in the prescription of treatment plans for patients with OUD. Scoping reviews are conducted over systematic reviews when the main purpose of the review is to identify gaps in existing knowledge. In accordance with PRIMSA-ScR, the researchers’ goal was to examine the literature thematically in order to synthesize evidence and assess the scope of the literature on providers’ stigmas of OUD.^3^,^4^ A methodological framework developed by Arksey and O’Malley^5^ and expanded upon by Levac et al. was used.^6^ This included identifying and clearly defining the research question; searching for and identifying relevant studies; selecting studies; charting the data both quantitatively and thematically; and summarizing the results.

### Inclusion Criteria

The following inclusion criteria were used for study inclusion: (1) a focus on patients with OUD; (2) a focus on stigmas and bias from healthcare practitioners; (3) based in Appalachia; (4) published in a peer-reviewed journal; (5) published between 1999 and 2021; and (6) limited to the English language, given the Appalachian regional focus of this review.

### Searching For and Identifying Relevant Studies

A comprehensive search strategy was developed through an iterative and collaborative process by a medical librarian. Searches were performed in MEDLINE PubMed, PsycInfo (EBSCOhost), CINAHL with Full Text (EBSCOhost), and Web of Science (Clarivate). The coverages of each database, as well as other essential information for search reproducibility and adherence to PRISMA Extension for Searching, can be found in the study’s protocol.^4^ Search terms were selected through an initial scan of the literature using MEDLINE PubMed and sentinel articles.^4^ The initial literature search was conducted in PubMed MEDLINE and translated using Polyglot^7^ in SR-Accelerator.^8^ The full search strategies and limits used can be found in the Additional Files section.

**Figure 1:**
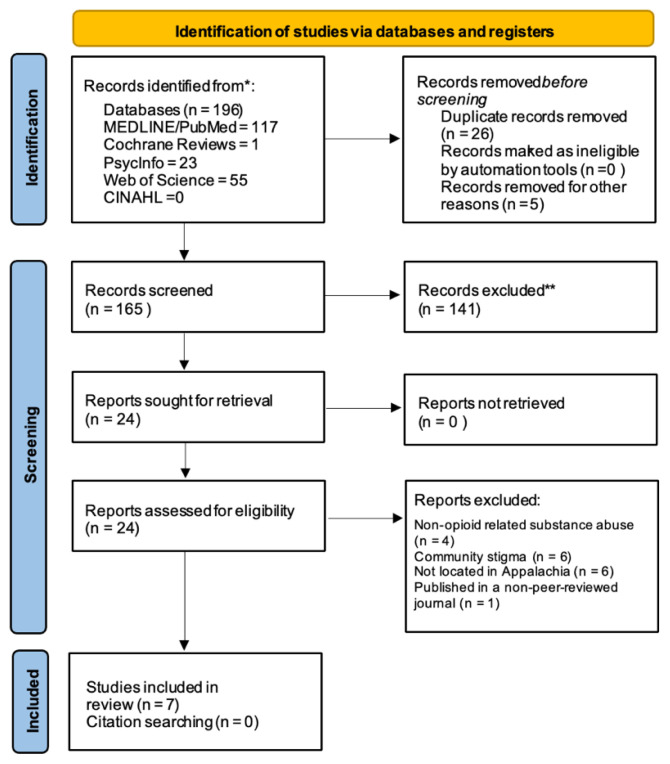
PRISMA 2020 flow diagram for new systematic reviews which included searches of databases and registers only NOTE: This diagram was adapted from Page MJ, McKenzie JE, Bossuyt PM, Boutron I, Hoffmann TC, Mulrow CD, et al. The PRISMA 2020 statement: an updated guideline for reporting systematic reviews. BMJ 2021;372:n71.

Searches were conducted on December 16, 2021, and deduplicated with SR-Accelerator. 8 To ensure this scoping review captured the full extent of the literature, hand searching of included studies’ reference lists was also completed. A PRISMA flowchart summarizing search strategy is described in Fig. 1. While citations extracted included articles from 1999 to 2021, the final included articles were only from 2019 to 2021, demonstrating that this is a relatively new area of research.

### Analysis

Data from the included articles was distilled and charted. Two individual coders first completed the title-abstract review, and a full-text review for articles considered for inclusion followed. These coders discussed any discrepancies and finalized the remaining studies to be included. The following data was extracted and charted: (1) author and year of publication; (2) research approach (quantitative, qualitative, or mixed method); (3) state and region of Appalachia, as defined by the *Health Disparities in Appalachia* report^9^; (4) type of healthcare provider; and (5) population of study (provider and provider–patient).

## RESULTS

### Populations of Study

All included articles were published between 2019 and 2021, highlighting the recent interest in this topic. Most included studies focused solely on the provider’s perspective (71.4%) except for two studies that integrated both provider and patient perspectives (28.6%). Only one article focused solely on physicians (14.4%) while the remainder was an even split between pharmacists (42.8%) and interdisciplinary providers (42.8%).[Table t1-jah-4-3-87]

### Research Methods

Research approach was evenly split between a quantitative (42.8%) and qualitative (42.8%), with one mixed-method study (14.4%). Quantitative studies used surveys, while qualitative studies primarily had in-depth interviews. The mixed-method study included with both quantitative and qualitative questions.

### Identified Themes

Three main themes were identified in the literature: (1) rural–urban differences in bias; (2) provider concern regarding legal implications and regulatory issues; and (3) the belief that OUD is a moral failing rather than a medical diagnosis. The primary outcome of each included study was distilled and charted (Table 2). Thematic subcategories were derived from analyzing the data and outcomes of each study.

#### Theme 1: Rural–Urban Differences

Four studies identified increased bias in rural settings when compared to an urban counterpart. Franz et al.^10^ identified a moderate level of bias amongst all surveyed physicians with a statistically significant higher amount of bias from physicians in rural counties. Substantiating this rural–urban difference, researchers accounted for factors known to increase bias and found no significant differences in the amount of physician contact with patients who misused opioids, stress related to working with this particular patient population, burnout levels, or physician specialty.^10^ This supports the presumption that patients with OUD will experience greater prejudice from their healthcare provider in a rural setting. Building on this assertion, nearly 50% of pharmacists surveyed in Alabama believed that naloxone allows people to continue using opioids at a riskier level than they would without naloxone, with a statistically significant increase in of this belief in rural pharmacies.^11^

Attempting to decode this rural–urban difference, researchers found that some providers blamed a “conservative” and “Appalachian” culture with low tolerance for illicit drugs as the main contributor to the belief that drug use is a moral issue to be addressed in the criminal justice system rather than a medical disease.^12^ The diverse group of interdisciplinary providers interviewed by Richard et al.^12^ explained that within this culture, medication for OUD (MOUD) is viewed to be as harmful as illicit opioids and that the use of medication-assisted treatment (MAT) is simply trading one addiction for another. It is clear this MOUD-related stigma permeates Appalachian culture and is exacerbated in a healthcare setting.

#### Theme 2: Legal Implications

Due to criminalization of substance misuse, particularly opioid misuse, many providers fear legal repercussions with MOUD. Two studies found that pharmacists were less likely to dispense MOUD due to lack of knowledge regarding naloxone dispensing laws.^13^, ^14^ Another study investigating naloxone-dispensing practices reported that most pharmacists stated that a lack of knowledge regarding naloxone-dispensing laws was as a major barrier to dispensing naloxone.^14^

In addition to a fear of legal repercussion, pharmacists may be discouraged by their distributors from stocking the medications. In one interview, a pharmacist disclosed the reasoning behind their regulatory concern with dispensing MOUD to Ostrach et al.: “The wholesalers said to them, ‘You don’t wanna open that can of worms. You don’t wanna mess with it. You don’t wanna deal with it.’”^15^

These stocking and dispensing issues are not the sole reason some pharmacists refuse to fill MOUD prescriptions.^15^ Ostrach et al.^15^ were informed of patient experiences in which a pharmacist would tell a patient over the phone that they had buprenorphine in stock and would dispense to them but would refuse when the patient arrived. As a result, it is not uncommon for patients to wish they had never initiated MAT, with some patients even advising their peers to avoid MAT in order to prevent stigmatizing experiences and withdrawal symptoms from pharmacy-related dispensing delays.^15^ This further fuels the perception of OUD as a moral failing rather than a treatable disease, the third theme in the literature.

#### Theme 3: OUD as a Moral Failing

All seven articles identified moral and ethical concerns from providers regarding opioid misuse. Self-awareness from some providers was noted, and these expressed that there is a fine line between demonstrating their “moral outrage” and maintaining professionalism.^16^ However, opioid-related stigma is not limited to the traditional patient–provider interaction, and it has been shown to infiltrate all aspects of health care.

Even before the first appointment for MAT, provider stigma can be embedded in insurance billing and pervasive cash practices, with some physicians charging out-of-pocket office fees as high as $600 per month.^16^ This form of structural stigma, which quickly shifts to enacted stigma, often allows healthcare providers to commodify patients with OUD in an opportunity to make their clinics money, instead of providing true patient-centered care.

Expanding on the notion opioid-related stigma is present in all healthcare settings, Ostrach et al.^15^ affirmed that most of their study participants with OUD reported stigma from pharmacy staff when attempting to have an MOUD prescription filled. A negative pharmacy experience seemed so normal to patients that when a positive pharmacy experience occurred, it was as shocking as it was appreciated.^15^ One patient shared value of their positive experience, “It means a whole lot [to have it go smoothly] because you can feel kind of ashamed. . . . People do look at you like, ‘oh you must be a drug addict,’ that sort of thing. That really means a lot to just be treated normal. . . . It means the world that these pharmacists are willing to treat me like anybody else.”^15^

Another unique finding related to MOUD-related stigma is that many pharmacists report infrequently dispensing MOUD and naloxone despite having filled prescription opioids for the same patient in the past.^13^ A scenario-based question in the same study revealed that less than half of MOUD and naloxone prescriptions would be dispensed by the pharmacists surveyed.^13^ The implication of this discovery supports the conjecture that MOUD and naloxone are viewed as unnecessary medications for a moral failing rather than crucial treatments for OUD.

While most of pharmacists Thompson et al.^14^ surveyed reported little to no barriers with dispensing naloxone, less than 25% of them had actually done so in the past year. Many of these pharmacists reported being comfortable dispensing naloxone only to patients who did not purchase syringes, indicating a difference in their stance on supplying naloxone when it is used for illicit drug overdose as compared to a prescription pain medication overdose.^14^ This suggests a potential disconnect between a provider’s conscious stance on dispensing naloxone and their unconscious sentiment.

## DISCUSSION

The people of Appalachia are no strangers to troubled healthcare access, consistently facing immense poverty, low health literacy, and inadequate insurance coverage.^1^ The additive effect of these barriers, combined with stigma and bias from healthcare providers, has created an enormous health disparity in the region. Research focused on provider bias toward OUD in Appalachia is limited, and this scoping review was conducted to address the gaps.

In this scoping review, only seven primary studies were identified that fit the inclusion criteria. The results indicate a paucity of research focused specifically on enacted provider bias toward patients with OUD in Appalachia. In these seven studies, three main themes emerged: (1) rural–urban differences in bias; (2) provider concern regarding legal implications and regulatory concerns; and (3) the belief that OUD is a moral failing rather than a medical diagnosis.

Rural–urban differences in provider bias were illuminated, as rural physicians reported higher levels of bias toward patients with OUD than their urban counterparts.^10^ The finding of provider stigma being correlated with rurality implies that residents of Appalachia face significantly greater bias when accessing medical care compared to patients in urban areas. In the past, interventions to reduce opioid-related stigma have focused on the three key predictors of bias: the amount of contact with patients with OUD, stress related to this contact, and burnout.^10^ However, these variables did not vary systematically between physicians in urban and rural counties, suggesting that there is something greater at play.^10^

To uncover the reason behind this rural–urban difference, it is important to investigate the differences between these environments. Certain aspects of Appalachian culture, including religion and local politics, could be confounds in the problematic view of opioid addiction. It would be impossible for rural providers to be completely immune to the pro-abstinence, “conservative” culture of Appalachia, and it is likely that rural providers are influenced by the opinions of their communities. Personal bias cultivated by Appalachian culture and fear of repudiation from the community for supporting patients with OUD bolsters opioid-related stigma and a lack of MAT utilization. Classical bias-reduction interventions may not be effective amongst rural healthcare providers due to the strong cultural influence of their communities.

Increasing social contact outside of the hospital may improve providers’ attitudes toward patients with OUD.^17^ For example, Corrigan et al.^17^ found that hosting community events highlighting MAT success stories could reduce MAT-related stigma and potentially improve patient–provider interactions. Assuming that rural–urban provider differences are the result of cultural influences, it could be inferred that a community event of this nature would be most beneficial in rural areas.

Yet improving one provider’s perception will not solve the entire problem. Even when prescribers confront their biases and practice evidence-based medicine, their patients may face discrimination in other healthcare settings, like a pharmacy, adding to the fear and mistrust many patients already have toward the healthcare system. In a study in Tennessee, less than 40% of pharmacists believed that the pain management and MOUD prescribers in their area practiced evidenced-based medicine.^13^ This widely held, harmful stigma regarding MAT is likely one of the main reasons many pharmacists refuse to fill MOUD prescriptions. The same pharmacists who hold negative opinions of MAT may be less likely to dispense naloxone, which could be investigated in future research.

Many pharmacists were recently granted the right to dispense naloxone without a prescription to combat the effects of the opioid epidemic.^18^ However, only 25% of pharmacists Thompson et al.^14^ surveyed had actually dispensed naloxone in the past year. Implementing techniques that reduce pharmacist stigma can play a pivotal role in improving health outcomes for patients with OUD. A common way to reduce pharmacists’ enacted stigma is to implement dispensing protocols with local MOUD prescribers and treatment centers.^15^ Patients who visited pharmacies with pre-arranged MOUD-dispensing protocols experienced less stigma than at other pharmacies they visited.^15^ This information provides valuable insight into practical ways to improve patients’ experiences and reduce stigmatizing pharmacy experiences that could act as a deterrent to MAT.

Lastly, many residents of Appalachia face a shortage of addiction treatment centers and harm reduction services, likely due to provider stigma. MAT-related stigma affects both treatment uptake and utilization, suggesting that MAT-related stigma becomes enacted as an unavailability of MAT providers in rural regions.^19^ The lack of support for medical treatment for OUD, in both healthcare and social settings, prevents the adequate health care desperately needed in Appalachia. Opioid-related stigma is broadly embedded in medical and social institutions in Appalachia, and all aspects must be addressed to improve region-wide health outcomes.

### Limitations

This scoping review has limitations, including the heterogeneity of studies, participant self-selection and social desirability bias, and the absence of a clearly defined operational definition for opioid-related provider bias. The articles included reflect the perspectives of providers willing to participate in a survey and people who have used opioids and felt comfortable participating in a research study about OUD. Additionally, participants may have intentionally answered in a desirable manner, skewing their true level of bias. At the time of creation for this scoping review, the research question formulated was, “What are providers’ stigmas and prescribed treatment plans for those with OUD in Appalachia?” However, after evaluating the literature, it became apparent that there was not a clearly defined operational definition of provider stigma in any study. The operationalization of provider stigma in our literature was potentially imprecise and could have led to assumptions regarding provider stigma based on the survey questions.

## IMPLICATIONS

The discoveries of this scoping review are extremely important because the same communities most affected by the opioid epidemic are also experiencing significant bias from their healthcare providers. These findings should provide guidance for future research aimed at creating a holistic understanding of provider bias towards patients with OUD in Appalachia. Future studies should examine providers’ treatment plans for OUD patients, scrutinizing the embedded stigma. Future studies should also target concept saturation, incorporating both provider and patient perspectives on opioid-related stigma in health care in Appalachia.

The implication of this scoping review is that there is a need for more studies that clearly demonstrate the impacts of provider bias and stigma on health-related outcomes and prescribed treatment plans. More research studies will also be necessary to ultimately conduct a systematic review and meta-analysis that can assess the magnitude of specific enacted stigmas.

Finally, recommendations for decreasing enacted provider opioid-related stigma may include hosting community events that feature MAT success stories, implementing pre-arranged MOUD dispensing protocols at all pharmacies, and increasing provider trainings that describe the current MOUD legislation in that area. Additionally, physicians from rural areas are most likely to establish practice in rural areas, suggesting that Appalachian healthcare training facilities should be integrating this type of evidence-based medicine into their curriculum.^20^ Appalachians deserve equitable health care, and it is crucial that the proper measures are taken now to improve future safety of people who use opioids.

**SUMMARY BOX**

**What is already known about this topic?**

Isolated research studies with varied designs have focused on the relationship between Opioid Use Disorder (OUD) and provider stigma. Yet on their own, these are insufficient in understanding the totality of enacted opioid-related stigma.

**What is added by this report?**

This scoping review draws common themes across studies in the existing literature and highlights the scarcity of research focused on the effects of provider stigma toward patients with OUD.

**What are the implications for future research?**

Through this review, a clear need emerges for more studies that clearly demonstrate the impacts of provider bias and stigma on health-related outcomes. Future studies should examine providers’ OUD treatment plans for embedded bias and incorporate both provider *and* patient perspectives on stigma.

## Supplementary Information





## Figures and Tables

**Table 1 t1-jah-4-3-87:** Characteristics of included studies (N = 7)

Study characteristics	n	%
**Publication year**		
2019	2	28.6
2020	2	28.6
2021	3	42.8
**Location**		
***State***		
Alabama	1	14.3
Ohio	4	57.1
North Carolina	1	14.3
Tennessee	1	14.3
***Region of Appalachia***		
North Central	2	28.55
South Central	1	14.3
Southern	1	14.3
South Central & Central	1	14.3
North Central & Central	2	28.55
**Type of Healthcare Professional**		
Physician	1	14.4
Pharmacist	3	42.8
Interdisciplinary Providers	3	42.8
**Research Approach**		
Quantitative	3	42.8
Qualitative	3	42.8
Mixed method	1	14.4
**Populations of Study**		
Provider	5	71.4
Provider & Patient	2	28.6

**Table 2:** Outcomes of included studies (N = 7)

Authors	Year	Approach	Healthcare Professional	Primary Outcome
Franz et al.	2021	Quantitative	Physician	Rurality is positively associated with physician bias toward patients who misuse opioids, independent of key predictors of bias including addiction specialties and the presence of harm reduction resources.
Ostrach et al.	2021	Qualitative	Interdisciplinary Providers	Patients with OUD experience increased stigmatizing treatment at rural, community pharmacies when attempting to fill MOUD.
Richard et al.	2020	Qualitative	Interdisciplinary Providers	Healthcare providers are affected by the culture of rural regions causing OUD to be treated as a moral/criminal problem rather than a medical issue.
Salwan et al.	2020	Quantitative	Pharmacist	The limited discussion of naloxone could be related to a lack of knowledge of the current laws surrounding naloxone dispensing in addition to bias.
Sisson et al.	2019	Quantitative	Pharmacist	Over 80% of pharmacists endorsed at least one negative belief about naloxone. However, pharmacists at rural, independent pharmacies were more likely to hold this belief than their urban counterparts.
Syversten et al.	2021	Qualitative	Interdisciplinary Providers	Evidence of stigma emerged across multiple healthcare contexts in this study. Structural stigma created barriers to care via insurance practices and punitive drug treatment while enacted stigma manifested as mistreatment from healthcare providers.
Thompson et al.	2019	Mixed Method	Pharmacist	The pharmacists surveyed stated that lack of knowledge on current Ohio laws was one of the greatest barriers to dispensing naloxone. However, there were significant moral/ethical concerns cited by providers.
