# Supplementary material for: Providers’ Stigmas and the Effects on Patients with Opioid Use Disorder: A Scoping Review
Source: J Appalach Health. 2023 Jan 1;4(3):87–102. doi: 10.13023/jah.0403.06 (PMC10655729; doi:10.13023/jah.0403.06)
Supplement: Supplementary file 2 [file 4.3.6_Skaggs_Additional_File_2.pdf]

PROTOCOL

Title: A Scoping Review Examining Providers' Stigmas and the Effects on Patients with Opioid Use Disorder: A Protocol

Authors: Skaggs, Peyton; F. Douglas Scutchfield; Robinson, Lauren E.; Bell, Sarah Beth.

Institution: University of Kentucky

CONTRIBUTION

The primary researcher (PS) formulated the premise for this systematic review. All four authors designed and drafted this protocol. The three subject researchers identified the research question and designed study selection and analysis process. The medical librarian built and conducted the search strategy, as well as developed the data management strategy. All authors made substantive intellectual contributions to the development of this protocol, and all have read and approved the manuscript.

INTRODUCTION

Bias and stigmas in healthcare have been a hot topic over the past couple of years. Diversity and inclusion boards have popped up at nearly every academic institution and hospital. Race, gender, sexuality, and disabilities have been at the center of every conversation. However, one of the most prevalent and critical stigmas remains unaddressed. The lack of scrutiny regarding this tangible bias is a missed opportunity for truly improving healthcare in Appalachia.

Stigmas in healthcare shape the access and quality of care patients receive. Opioid use stigma is a complex concept that further perpetuates the opioid epidemic. To improve this disparity, it is essential to examine the literature and understand the evidence provided by these studies. Unfortunately, research on the relationship between opioid use disorder and provider stigma is greatly lacking which has created the need for this in-depth analysis.

Researchers will investigate the evidence available in the peer-reviewed literature in order to better understand the ways provider bias manifests into enacted stigma in Appalachia. We are particularly interested in research in Central Appalachia (Tennessee, Kentucky, West Virginia, and Virginia), as the region is a hub for the opioid epidemic. The objective of this scoping review is to call attention to the egregious disparities in healthcare affecting those with opioid use disorder.

STUDY OBJECTIVES

The objectives of this review are as follows:

- Analyze and summarize the results of peer-reviewed literature examining the stigma associated with Opioid Use Disorder and how these stigmas affect providers' prescribed treatment plans.

- Categorize the effects of each factor and investigate any developing patterns.
- Provide recommendations to decrease the social stigma associated with Opioid Use Disorder and prevent any bias in providers' treatment plans.
- Determine areas in which further research is necessary.

METHODS AND ANALYSIS

The researchers will conduct a scoping review to examine the literature on how health care providers' stigmas affect prescribed treatment plans in those with Opioid Use Disorder. In particular, the researchers aim to focus on literature in the Appalachian region of the United States. The researchers investigating this question are meant to thematically examine the literature in order to understand the connection between providers' stigmas of opioid use disorder and provider choice and patient care.

The researchers chose a scoping review methodology because of its goal to "map evidence on a topic and identify main concepts, theories, sources, and knowledge gaps" within the published literature [3]. The researchers will use the methodological framework developed by Arksey and O'Malley and expanded upon by *Levac et al.* [4 5]. This method will include (A) identifying and clearly defining the research question; (B) searching and identifying relevant studies; (C) study selection; (D) charting the data both quantitatively and thematically; and (E) summarizing and reporting the results.

Defining the Research Question

Since scoping reviews require addressing a broad research question in order to synthesize the breadth of the literature, it is important to clearly define the variables that will be searched. Through consultation and iterative revisions, the research team developed one broad research question: ***What are providers' stigmas and prescribed treatment plans for those with opioid use disorders in Appalachia?*** Consistent with the Levac et al model, researchers have defined and operationalized study concepts and target populations. For this review, opioid use disorders will be defined as disorders related to or resulting from abuse or misuse of opioids. Synonyms will include terms such as opioid, opium, morphine, heroin, and substance abuse. Stigma will be defined as a perceived attribute that is deeply discrediting and is considered to be a violation of social norms (MeSH). Stigma will include but will not be limited to terms such as social stigma, bias, prejudice, attitudes, and marginalization. Health care providers will be defined as those working in the provision of health services, whether as individual practitioners or employees of health institutions and programs, whether or not professionally trained, and whether or not subject to public regulation (MeSH). This will include terms such as health personnel, health worker, practitioner, health professional, dentist, physician, doctor, clinician, therapist, pharmacist, and provider. Finally, Appalachia is defined as the geographical area of the United States which includes portions of Alabama, Georgia, South Carolina, North Carolina, Kentucky, Tennessee, West Virginia, Virginia, Maryland, Pennsylvania, Ohio, and New York (MeSH).

Searching and Identifying Relevant Studies

A comprehensive search strategy will be developed through an iterative and collaborative process among the researchers and the medical librarian. Relevant studies will be identified through searching MEDLINE PubMed, PsycInfo (EBSCOhost), CINAHL with Full Text (EBSCOhost), and Web of Science (Clarivate). The coverages of each database are described in Table 1. The researchers selected these databases based on institutional availability, alongside the breadth of discipline coverage, which spans healthcare, public health, and social sciences disciplines. These sources will be searched using a combination of relevant search terms and subject headings that were developed through an initial scan of the literature using MEDLINE PubMed and sentinel articles. The terms, detailed in Table 2, will be adapted to match the syntax of each electronic database. To ensure this scoping review captures the full extent of the literature, extensive hand searching of reference lists will be completed on all included studies. Finally, according to the National Association of Counties (NACo) AND Appalachian Regional Commission (ARC) white paper, "Opioids in Appalachia The Role of Counties in Reversing a Regional Epidemic" the opioid epidemic began in 1999 (NACo & ARC, 2019). Therefore, the search will include a date limit from 1999 to present.

Search Strategy Data Management Plan

The medical librarian will create a table recording essential information, including but not limited to date searches were conducted, search strategies, and number of search results. The resulting citations will be exported to EndNote Citation Management Software. The Groups function will be used to sort references by database. Since the Groups function keeps a record of citations, this process will act as a backup to the table mentioned previously. The EndNote library will be copied, and the deduplication function of EndNote will be used to reduce the number of citations to review. Both EndNote libraries will be saved as individual files in order to maintain a record of the deduplication process and reduce overall error. After the initial title/abstract review has been completed, the medical librarian will pull and attach PDFs to the EndNote Library so full-text review can be completed. In order to ensure continuous access, the medical librarian will keep remote copies of all files using OneDrive.

Study Selection

Inclusion and Exclusion Criteria

The following inclusion criteria will be used to lead the search and will also be used to review articles: (1) The study focuses on patients with Opioid Use Disorder; (2) The study focuses on stigmas and bias from healthcare practitioners; (3) The study is based in Appalachia; (4) The study is published in a peer-reviewed journal; (5) The study was published between 1999 – 2021; and (6) Because of the Appalachia regional focus of this review, literature will be limited to English language.

The exclusion criteria that have been identified are: (1) The study focuses on non-opioid related substance abuse; (2) The study focuses on community stigma; and (3) The study was published prior to 1999, the year indicative of the opioid boom in Appalachia.

Study Selection Process
Review of articles will be carried out in a synchronous process. Decisions on criteria and interpretation of outcomes will be made by the lead researcher in collaboration with the assistant researcher. Since the nature of a scoping review is to collate, synthesize, and describe the coverage of the literature, no assessment of quality will be made but the review will assess whether the study achieved its intended outcomes. The medical librarian will lead the process and source identification for the scoping review. To ensure the standards of a scoping review are upheld, the medical librarian will conduct the searches and provide the results to the senior researcher and research assistant using EndNote. Deduplication will occur using both EndNote and SR-Accelerator Deduplicator (Rathbone, et al., 2015). After deduplication, the team will screen the remaining titles using the stated inclusion and exclusion criteria. The first step of screening will be at the title/abstract level. After title/abstract review, the medical librarian will retrieve full-text of included studies. The lead researcher and research assistant will then conduct full text screening using the inclusion and exclusion criteria. If a disagreement is encountered, the lead researcher and research assistant will discuss and decide whether an article should be included through a review of the larger themes and ideas of the article. Furthermore, inclusion and exclusion criteria will be updated through holistic discussions among the team.

Charting the Data

Excel will be used to assemble and chart data throughout the research process. The first phase will be title-abstract review. The researchers will collectively review title abstracts for inclusion and exclusion criteria. The second phase will be full-text review. The lead researcher and research assistant will simultaneously extract data from the same five randomly selected studies. Reviewing the same articles ensures clarity and enables both researchers to synthesize separate findings into one conclusion. This approach is the most efficient and will limit the amount of time spent reviewing multiple, potentially unnecessary articles. This norming process will follow the title-abstract review and will include the lead researcher and assistant researcher. In order to assure quality and replicability, the lead researcher and assistant researcher will meet to examine each other's extractions. During this meeting, the researchers will come to agreement on extraction methods and details. Afterwards, the remaining included studies will be divided, and extraction will continue until completed.

The data that will be extracted will include:
- Author, year of publication, and other background information;
- Research approach (quantitative, qualitative, mixed methods, etc.);
- State and region of Appalachia as defined by the Health Disparities in Appalachia report (Marshall et al., 2017);
- Type of healthcare professional

To adequately describe the characteristics of the literature, additional quantitative and qualitative data points may be added by the researchers throughout the process of data extraction and charting.

Summarizing and Reporting Results

FINAL SEARCH STRATEGY

(((((((((((((("Health Personnel"[Mesh]) OR ((healthcare[tiab] OR "health care"[tiab] OR health[tiab]) AND (personnel*[tiab] OR worker*[tiab] OR profession*[tiab] OR practitioner*[tiab] OR provider*[tiab]))) OR ("Physicians"[Mesh] OR Physician*[tiab] OR doctor*[tiab])) OR ("Dentists"[Mesh] OR Dentist*[tiab] OR Periodontist*[tiab] OR Prosthodontist*[tiab])) OR (("Nurses"[Mesh] OR "Nurse Midwives"[Mesh]) OR (Nurse*[tiab] OR Nursing[tiab] OR midwife*[tiab] OR midwives[tiab]))) OR (("Pharmacists"[Mesh]) OR (Pharmacist*[tiab] OR Chemist*[tiab]))) OR (Clinician*[tiab])) OR (Therapist*[tiab])) OR ("Social Workers"[Mesh] OR "social worker"[tiab] OR "social workers"[tiab] OR "socialworker"[tiab] OR "socialworkers"[tiab])) OR ("public health worker"[tiab] OR "public health workers"[tiab])) AND ("Social Stigma"[Mesh] OR "Attitude of Health Personnel"[Mesh] OR stigma*[tiab] OR bias[tiab] OR biases[tiab] OR prejudice*[tiab] OR marginaliz*[tiab] OR attitude*[tiab])) AND (("Substance-Related Disorders"[Mesh:noexp] OR "Opioid-Related Disorders"[Mesh] OR "Opium Dependence"[Mesh] OR "Heroin Dependence"[Mesh] OR "Morphine Dependence"[Mesh]) OR ((substance[tiab] OR prescription*[tiab] OR drug*[tiab] OR "Heroin"[Mesh] OR heroin*[tiab] OR Diacetylmorphine[tiab] OR Diamorphine[tiab] OR Diamorf[tiab] OR "Morphine"[Mesh] OR morphine*[tiab] OR morphia[tiab] OR "SDZ 202 250"[tiab] OR contin[tiab] OR oramorph[tiab] OR duramorph[tiab] OR "Opium"[Mesh] OR opium*[tiab] OR "Analgesics, Opioid"[Mesh] OR opiate*[tiab] OR opioid*[tiab] OR fentanyl*[tiab] OR Fentanest[tiab] OR Fentora[tiab] OR Duragesic[tiab] OR Sublimaze[tiab] OR Phentanyl[tiab] OR R4263[tiab] OR "R 4263"[tiab] OR "Narcotics"[Mesh] OR narcotic*[tiab]) AND (abuse*[tiab] OR use[tiab] Or uses[tiab] OR usage[tiab] OR disorder*[tiab] OR addiction*[tiab] OR dependence*[tiab] OR habit*[tiab]))))) AND (("Appalachian Region"[MeSH Terms] OR "appalachia*"[tiab]) OR (("appalachia*"[tiab] OR "Rural Population"[MeSH Terms] OR "rural*"[tiab]) AND (Alabama[MeSH] OR Georgia[MeSH] OR Kentucky[MeSH] OR Maryland[MeSH] OR "New York"[MeSH] OR "North Carolina"[MeSH] OR Ohio[MeSH] OR Pennsylvania[MeSH] OR "South Carolina"[MeSH] OR Tennessee[MeSH] OR Virginia[MeSH] OR "West Virginia"[MeSH] OR Alabama*[tiab] OR Georgia*[tiab] OR Kentucky[tiab] OR Kentuckian[tiab] OR Maryland*[tiab] OR "New York*"[tiab] OR "North Carolina*"[tiab] OR Ohio*[tiab] OR Pennsylvania*[tiab] OR "South Carolina*"[tiab] OR Tennessee*[tiab] OR Virginia*[tiab] OR "West Virginia"[tiab])))) AND ((("1999/01/01"[Date - Create] : "3000"[Date - Create])))

Table 1: Database Access and Coverage

DATABASE	VENDOR	COVERAGE START
Medline PubMed	NIH	1966

CINAHL with Full Text	EBSCO	1963
PsycINFO	EBSCO	Extensive coverage from 1800s to the present
Web of Science Core Collection	Clarivate	Sciences: 1900-present<br>Social Sciences: 1900-present<br>Arts & Humanities: 1975-present
Cochrane Database of Systematic Reviews	Wiley	April 1996

(Clarivate, 2021; EBSCOhost (n.d.); National Library of Medicine, 2021; Wiley (n.d.);)

Table 2: ***Term Mapping and Selection***

**Health Personnel**	Health personnel<br>Healthcare worker<br>Health professional<br>Healthcare professional<br>Health worker<br>Health care personnel<br>Health practitioner<br>Health care provider<br>Public Health workers	Physician<br>Doctor<br>Dentist<br>Dental<br>Nurse<br>Clinician<br>Therapist<br>Pharmacist<br>Social worker
**Substance-Related Disorders**	Substance Abuse<br>Substance Use<br>Substance Related Disorder<br>Substance Dependence<br>Drug abuse<br>Drug Addiction<br>Drug Dependence<br>Drug Habituation<br>Prescription Abuse<br>Prescription Drug Abuse<br>Narcotics<br>Opiate<br>Diacetylmorphine<br>Diamorphine<br>Diamorf	Opioid<br>Heroin<br>Morphine<br>Morphia<br>Contin<br>Oramorph<br>Duramorph<br>Opium<br>Fentanyl<br>Phentanyl<br>Fentanest<br>Fentora<br>Duragesic<br>Sublimaze<br>R4263
**Social Stigma**	Attitude of Health Personnel<br>Stigma<br>Bias	Prejudice<br>Marginalization<br>Attitudes

REFERENCES

Clarivate. (2021). *Web of Science: Summary of Coverage.* Retrieved November 29, 2021, from https://clarivate.libguides.com/webofscienceplatform/coverage.

EBSCOhost. (n.d.). *APA PsycInfo*. Retrieved November 29, 2021, from https://health.ebsco.com/products/psycinfo.

EBSCOhost. (n.d.). *CINAHL Comparison Chart [PDF]*. Retrieved November 29, 2021, from https://www.ebsco.com/sites/g/files/nabnos191/files/acquiadam-assets/CINAHL-on-EBSCOhost-Comparison-Chart-Flyer.pdf

Madden, E. F. (2019). Intervention stigma: How medication-assisted treatment marginalizes patients and providers. Social science & medicine (1982), 232, 324–331. https://doi.org/10.1016/j.socscimed.2019.05.027

Marshall, J., Thomas., L, Lane, N., Holmes, G., Arcury, T., Randolph, R., Silberman, P., Holding, W., Villamil, L., Thomas, S., Lane, M., Latus., J., Rodger, J., and Ivey, K. (2017). Health Disparities in Appalachia. In: Commission AR, ed. Raleigh, NC: PDA, Inc., Cecil G. Sheps Center for Health Services Research, Appalachian Regional Commission. Retrieved from: https://www.arc.gov/wp-content/uploads/2020/06/Health_Disparities_in_Appalachia_August_2017.pdf

McCradden, M. D., Vasileva, D., Orchanian-Cheff, A., & Buchman, D. Z. (2019). Ambiguous identities of drugs and people: A scoping review of opioid-related stigma. The International journal on drug policy, 74, 205–215. https://doi.org/10.1016/j.drugpo.2019.10.005

National Association of Counties (NACo) and Appalachian Regional Commission (ARC). (2019). Opioids in Appalachia: The Role of Counties in Reversing a Regional Epidemic [White paper]. National Association of Counties (NACo). https://www.naco.org/sites/default/files/documents/Opioids-Full.pdf

National Library of Medicine. (2021). M*EDLINE®: Description of the Database: National Library of Medicine*. Retrieved November 29, 2021, from https://www.nlm.nih.gov/bsd/medline.html.

Rathbone, J., Carter, M., Hoffmann, T., and Glasziou, P. (2015). Better duplicate detection for systematic reviewers: evaluation of Systematic Review Assistant-Deduplication Module. Syst Rev. 2015 Jan 14;4:6. doi: 10.1186/2046-4053-4-6. https://www.ncbi.nlm.nih.gov/pubmed/25588387

Surratt, H. L., Otachi, J. K., McLouth, C. J., & Vundi, N. (2021). Healthcare stigma and HIV risk among rural people who inject drugs. Drug and alcohol dependence, 226, 108878. https://doi.org/10.1016/j.drugalcdep.2021.108878

Syvertsen, J. L., Toneff, H., Howard, H., Spadola, C., Madden, D., & Clapp, J. (2021). Conceptualizing stigma in contexts of pregnancy and opioid misuse: A qualitative study with women and healthcare providers in Ohio. Drug and alcohol dependence, 222, 108677. https://doi.org/10.1016/j.drugalcdep.2021.108677

Wiley. (n.d.). *Content Coverage Date Range for Cochrane Library*. Cochrane Library. Retrieved November 29, 2021, from https://cochranesupport.wiley.com/s/article/content-coverage-date-range-for-cochrane-library.
